# Lesions within the head direction system reduce retrosplenial c-*fos* expression but do not impair performance on a radial-arm maze task

**DOI:** 10.1016/j.bbr.2017.10.026

**Published:** 2018-02-15

**Authors:** Seralynne D. Vann

**Affiliations:** School of Psychology, Cardiff University, 70 Park Place, Cardiff, CF10 3AT, UK

**Keywords:** Anterior dorsal thalamus, Head direction cells, Lateral mammillary nuclei, Rat, Spatial memory

## Abstract

The lateral mammillary nuclei are a central structure within the head direction system yet there is still relatively little known about how these nuclei contribute to spatial performance. In the present study, rats with selective neurotoxic lesions of the lateral mammillary nuclei were tested on a working memory task in a radial-arm maze. This task requires animals to distinguish between eight radially-oriented arms and remember which arms they have entered within a session. Even though it might have been predicted that this task would heavily tax the head direction system, the lesion rats performed equivalently to their surgical controls on this task; no deficit emerged even when the task was made more difficult by rotating the maze mid-way through testing in order to reduce reliance on intramaze cues. Rats were subsequently tested in the dark to increase the use of internally generated direction cues but the lesion rats remained unimpaired. In contrast, the lateral mammillary nuclei lesions were found to decrease retrosplenial c-Fos levels. These results would suggest that the head direction system is not required for the acquisition of the standard radial-arm maze task. It would also suggest that small decreases in retrosplenial c-Fos are not sufficient to produce behavioural impairments.

## 1 Introduction

The lateral mammillary nuclei form a critical part of the head direction system given the head direction signal in both the anterior dorsal thalamic nuclei [1], [2], [3] and postsubiculum [4] is dependent on the outputs from the lateral mammillary nuclei. However, the functional importance of the head direction system is still poorly understood and only a few studies have investigated the behavioural effects of selective lateral mammillary nuclei lesions [5], [6], [7]. From these behavioural studies, a fairly consistent finding is that lateral mammillary lesions can often leave spatial performance intact and when impairments are found they tend to be transient and milder than those found following lesions to other regions such as the hippocampus, anterior thalamic nuclei or mammillothalamic tract.

While lateral mammillary nuclei lesions do not seem to affect the use of individual landmarks to direct behaviour [7], they do impair the use of geometric cues, for example, the ability to distinguish distinct corners of a rectangle within a water-maze [6]. Two further studies have found rats with lateral mammillary nuclei lesions to be impaired on water-maze tasks. The first was a working memory version of the task where the rats had to learn a new platform position on each test session. The lesion rats were initially slower to locate the hidden platform than their surgical controls but by the final trial of each day the two groups performed equivalently. Not only did the lesion rats improve within a test session but they also improved across sessions and there was no evidence of any impairment by the end of training (i.e., days 8–12) [5]. A second study looked at performance on a reference memory task in the water-maze where the platform position remained the same across training. While there was no lesion-induced impairment on this task, the lesion rats were subsequently impaired when the platform position was placed in the opposite location within the pool [7]. Again, this may suggest that lateral mammillary lesions disrupt rapid spatial encoding but this impairment could also reflect poor cognitive flexibility.

In the present study, rats were tested on a working memory task in the radial-arm maze. This task was selected because it is highly sensitive to lesions of the anterior thalamic nuclei [8], [9] and postsubiculum [10]; it is, therefore, possible that performance on this task is dependent on the head direction network. Further indication that the head direction system might be important for this radial arm maze task comes from recent studies suggesting that directional information might facilitate the discrimination of radially oriented environments, by enabling animals to form distinct place cell representations [11], [12]. The radial-arm maze task in the present study required animals to visit all eight arms of the maze within a session without re-entering any previously visited arms. For this task animals have to remember which arms they have visited within the session but they also have to differentiate between the radial-arms, potentially using directional cues.

It has been previously shown that lateral mammillary nuclei lesions leave standard T-maze alternation intact [5], [6]. However, animals often use a number of different spatial cues to perform spatial memory tasks, including allocentric cues, directional cues, and intramaze cues (e.g. odour cues) [13], [14], [15]. When animals were prevented from using intramaze cues to perform the T-maze task, by running sample and test phases in adjacent mazes [7], a lateral mammillary lesion impairment emerged. Considering these findings, it was important to carry out additional manipulations to encourage the use of allocentric and directional cues in the performance of the radial-arm maze task. The first manipulation involved rotating the maze mid-way through a trial to test the extent to which animals were using intramaze cues to perform the task. The second manipulation involved testing the animals in the dark, which should reduce reliability on allocentric cues and so encourage the use of directional cues.

Finally, lesions of the anterior thalamic nuclei produce widespread changes in distal brain structures as measured by the activity of the immediate-early gene c-*fos*. The changes are seen in sites such as the hippocampus proper and retrosplenial cortex [16], [17], [18], structures that are also important for memory. To determine the extent to which these anterior thalamic induced changes reflect the indirect loss of inputs from the lateral mammillary nuclei to the anterodorsal thalamic nuclei, expression of c-*fos* was assessed in both lateral mammillary nuclei lesion rats and their surgical controls shortly after they had performed a spatial task in a novel room.

## 2 Materials and methods

### 2.1 Animals

Nineteen male rats of the pigmented DA (Dark Agouti) strain (Harlan, Bicester, U.K.) were used in this study. All subjects were housed in pairs under diurnal conditions (14 h light/10 h dark). Food was restricted during behavioural testing but animals remained above 85% of their free-feeding weight throughout. Water was available *ad libitum* during testing. At the time of surgery, the animals were four months old and weighed 220–250 g. All experiments were performed in accordance with the UK Animals (Scientific Procedures) Act (1986) and associated guidelines. Some data from these animals have previously been published [6].

### 2.2 Surgery

Twelve rats underwent surgery for lateral mammillary lesions and seven rats were surgical controls. Animals were first anaesthetised with an intraperitoneal injection of sodium pentobarbital (60 mg/kg), and then placed in a stereotaxic headholder (David Kopf Instruments, Tujunga, CA), with the incisor bar set at +5.0 mm to the horizontal plane. An incision was then made in the scalp, and the skin retracted to expose the skull. A dorsal craniotomy was made directly above the target region and the dura cut to expose the cortex. The lateral mammillary lesions were made by injecting 0.30 μl of 63 mM ibotenic acid (Biosearch Technologies Inc., San Rafael, CA, USA) in phosphate buffered saline (PBS) at pH 7.2 into one site per hemisphere using a 1 μl syringe (Hamilton, Switzerland). The stereotaxic coordinates were as follows: antero-posterior, −2.2 mm from bregma; medio-lateral, ±1.0 mm from the midline; dorso-ventral, −9.2 mm from the top of the dura. The surgical controls underwent the same procedure with the needle being lowered but no injection was made. After every surgery, the skin was sutured together over the skull and antibiotic powder was applied to the wound (Acramide, Dales Pharmaceuticals, UK). All animals received 5 ml of glucose saline subcutaneously and were placed in a temperature-controlled recovery box. Paracetamol (for pain relief) and sucrose were added to the rats’ drinking water for three days post-surgery.

### 2.3 Radial-arm maze task

#### 2.3.1 Apparatus

Testing was carried out in an eight-arm radial maze. The maze consisted of an octagonal central platform (diameter 34 cm) with eight equally spaced radial arms (87 cm long, 10 cm wide) each with a recessed cylindrical food well (diameter 2 cm, 0.5 cm deep) at the end of each arm. The base of the central platform and the arms were made of wood, while clear Perspex formed the walls (24 cm high) of the arms. The central platform was placed on a clear, Perspex column (diameter 34.5 cm, 55 cm high). At the start of each arm was a clear Perspex sliding door (12 cm high) that controlled access in and out of the central platform. Each door was attached to a pulley system enabling the experimenter to control access to the arms from a distance. The maze was in a rectangular room that contained salient visual cues such as geometric shapes and high contrast stimuli on the walls.

Illumination during initial habituation and training in the ‘light’ was provided by two standard ceiling lights giving a mean illumination of 671 lx measured in the middle of the central platform. During training in the ‘dark’, the illumination was provided by a standard lamp, fitted with a 60W red light bulb placed inside the column of the radial-arm maze apparatus, immediately under the central platform. This arrangement for the ‘dark’ condition gave an average light intensity of 2.5 lx on the central platform. The room lights were switched off and a white cotton curtain was pulled around the outside of the radial-arm maze. The rats were transported in groups of four between the holding room and the radial-arm maze room in an opaque, aluminium travelling box.

#### 2.3.2 Procedure

Testing on the radial-arm maze task began approximately three months following surgery. In the intervening months, animals were tested on T-maze alternation and a geometric discrimination task in the watermaze [6]. Pre-training for the radial-arm maze involved three habituation sessions where the animals were allowed to explore the maze freely for 5 min. All the sliding doors were raised and reward pellets (45 mg; Noyes Purified Rodent Diet, UK) were scattered down the arms. This was followed by formal training, which lasted for 30 sessions and consisted of four stages.

The acquisition phase (sessions 1–15) was the standard working memory version of the radial-arm maze task [19] where the animals’ optimal strategy was to retrieve the reward pellets from all eight arms without re-entering any previously entered arms. At the start of a trial all eight arms were baited with a single reward pellet. The animal would make an arm choice and then return to the central platform and all the doors were closed for about 10 s before they were opened again, permitting the animal to make another choice. This procedure continued until all eight arms had been visited or 10 min had elapsed. To control for the use of intra-maze cues a maze rotation stage was carried out (Stage 2). For this, the maze was rotated by 45° (sessions 16–21) after the rat had made its first four arm choices. For the rotation, the rat was confined to the centre of the maze while the maze was rotated (either clockwise or anticlockwise), and the arms re-baited so that the spatial locations of the remaining pellets were unchanged with respect to the room cues. The trial then continued until all eight arms had been visited. Following this, the rats were moved to the original acquisition procedure for sessions 22–24 (Stage 3), i.e. rats were tested in the light using conditions identical to Stage 1. The final stage (sessions 25–30) assessed the lesion effects on maze learning in the dark. In all other aspects training was the same as acquisition in Stage 1, i.e. the maze was not rotated mid-trial.

### 2.4 Immediate-early gene study

#### 2.4.1 Apparatus

Two identical radial-arm mazes placed in two different rooms were used for the experiment. The two rooms (Room 1: 295 × 295 × 260; Room 2: 255 × 330 × 260) were markedly different (i.e. size, shape, lighting) and contained distinct, salient, visual cues such as geometric shapes and high-contrast stimuli on the walls. Room 1 was the same room in which the animals had previously been tested on the radial-arm maze task, whereas the animals had no previous experience of Room 2.

#### 2.4.2 Behavioural training

One week following completion of the radial-arm maze task, animals were trained to run down pre-selected arms of an eight-arm radial maze in Room 1 in order to retrieve sucrose reward pellets (45 mg; Noyes Purified Rodent Diet, Lancaster, NH, USA) from the end of the arm. On the final test day the animals performed the same task but in a novel room (Room 2). At the beginning of each trial all arms were baited; the experimenter controlled each arm the animals visited by using a pulley system to open the sliding door at the start of the arm. When the trial had been completed (i.e. all eight arms had been visited) the rat was contained in a holding box for approximately 2 min whilst all arms were re-baited. Each session consisted of multiple trials in the radial-arm maze, one after the other, and lasted 20 min. Different, randomised arm sequences were used on successive trials. Animals were run in matched groups that received the same amount of pre-training (mean number of trials ± standard error, 15.1 ± 0.58 over 5 or 6 days) and performed the same number of trials (3.0 ± 0.08) and visited the same arms in the same order the test day. Each animal was placed in a holding box in a quiet, dark room for 30 min before and 90 min after each radial-arm maze session. This time window was chosen as peak levels of the Fos protein are found approximately 90 min after activation [e.g. 20].

#### 2.4.3 Immediate-early gene immunohistochemistry

Ninety minutes after completing the final radial-arm maze session in the novel room, rats were deeply anaesthetised with sodium pentobarbital (60 mg/kg, Euthatal, Rhone Merieux, UK) and transcardially perfused with 0.1 M phosphate buffer saline (PBS) followed by 4% paraformaldehyde in 0.1 M PBS (PFA). The brains were removed and postfixed in PFA for 4 h and then transferred to 25% sucrose overnight at room temperature with rotation. Sections were cut at 40 μm on a freezing microtome in the coronal plane. One series (one-in-three sections) was collected in PBS. Sections were processed for c-Fos immunostaining using c-Fos rabbit polyclonal antibody (1:5000; Ab-5, Oncogene Science, UK). The methods have been described previously [31]. A second one-in-three series was mounted directly onto gelatine-coated slides and stained using cresyl violet, a Nissl stain, for verification of the lesion and histological identification of specific brain regions.

#### 2.4.4 Fos-positive cell counts

**Fig. 1:**
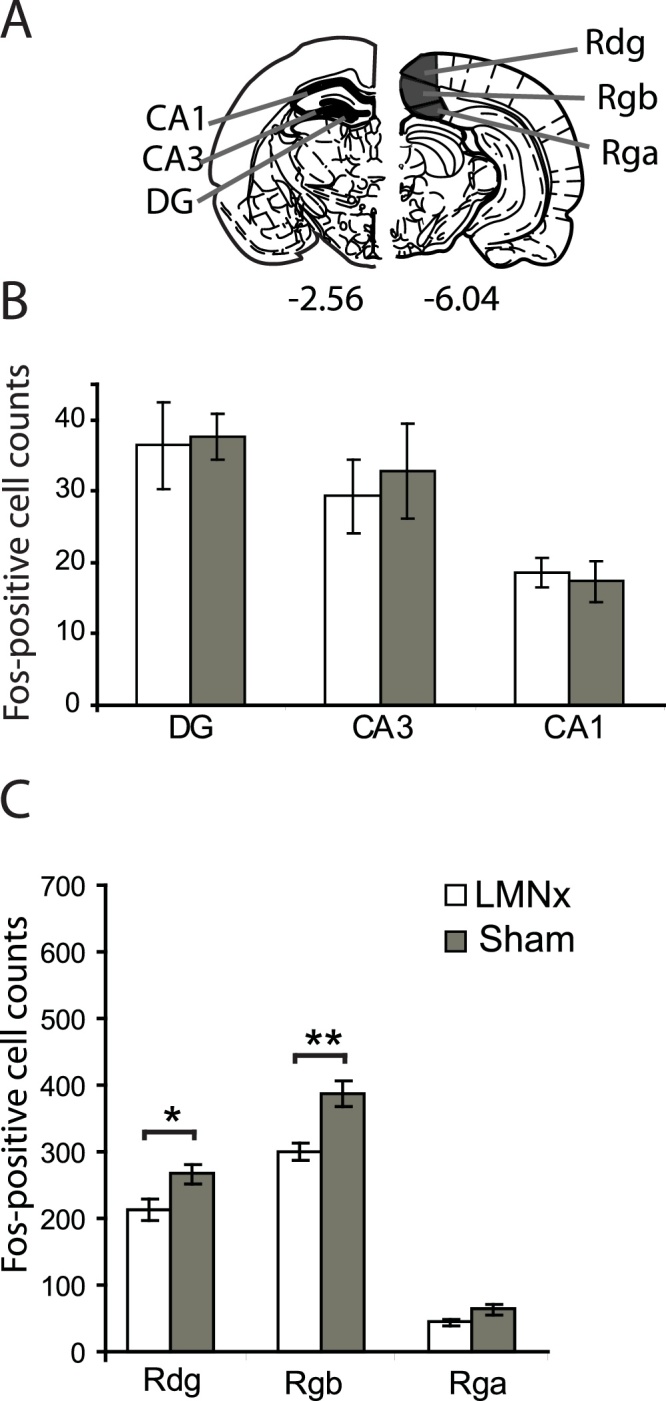
Immediate-early gene study A. Areas where c-Fos counts were taken for the hippocampus and retrosplenial cortex. The numbers represent the distance, in millimetres, from bregma. B. Mean number of Fos-positive cell counts for septal hippocampal subregions (dentate gyrus, CA3, CA1); C. Mean number of Fos-positive cell counts for retrosplenial cortex. Abbreviations: LMNx lateral mammillary lesion group; Sham, surgical control group; DG, dentate gyrus; Rdg, dysgranular retrosplenial cortex; Rgb, granular b retrosplenial cortex; Rga, granular a retrosplenial cortex. Error bars represent standard error of mean.*p ≤ 0.05, **p < 0.001.

Estimates were made of Fos-positive cells using an automated cell counting procedure. Counting procedures were carried out without knowledge of the group assignments. Images were captured using a Q Imaging MicroPublisher 3.3 RTV camera attached to a Zeiss Axiostar Plus microscope. The public domain NIH Image program (developed at the U.S. National Institutes of Health and available on the Internet at http://rsb.info.nih.gov/nih-image/) was used to count the number of nuclei above threshold. For hippocampal counts (dentate gyrus, CA3, and CA1), the entire extent of the target region within the selected coronal sections was assessed. These counting procedures were not stereological and so while providing information about relative numbers of cells they do not provide absolute counts of cell numbers. For each brain area analysed (Fig. 1a), counts were taken from at least four consecutive sections from each hemisphere and these counts were averaged to produce a mean.

##### 2.4.4.1 Regions of interest

Cytoarchitectonic subfields were identified from coronal sections [21], using the nomenclature of Swanson [22]. The regions sampled are depicted in Fig. 1a. The regions were specifically chosen either because they show changes in Fos expression following anterior thalamic lesions (i.e. the hippocampus proper and retrosplenial cortex).

**Fig. 2:**
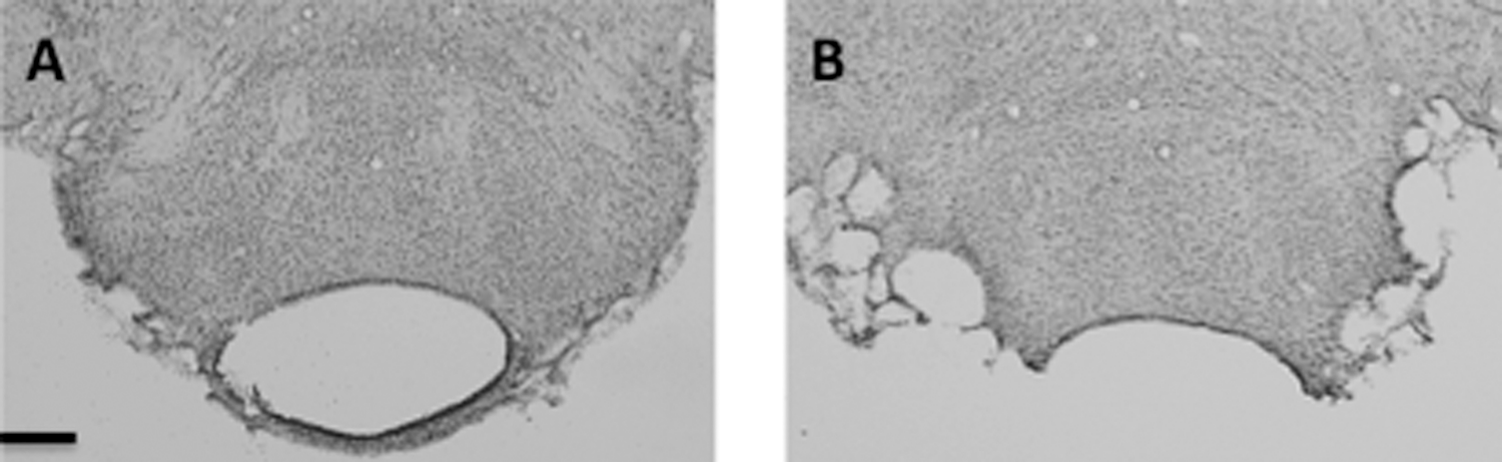
Photomicrographs of the lateral mammillary nuclei lesions. A. An example of the lateral mammillary lesion; B. Surgical sham. Scale bars: 0.25 mm.

Cytoarchitectonic subfields within the hippocampus consisted of dentate gyrus (DG), CA3, and CA1. Counts were taken from the septal pole of the hippocampus (Fig. 2). The retrosplenial cortex can be subdivided [23] into granular b cortex (Rgb), granular a cortex (Rga), and the dysgranular cortex (Rdg). Separate counts were made in all three retrosplenial subregions. The caudal region of the retrosplenial cortex was chosen as all retrosplenial subregions are present at this level (Rga is not present in anterior retrosplenial cortex). In terms of c-Fos, previous studies have typically found similar post-lesion changes along the anterior-posterior axis [24], [25], [26].

### 2.5 Statistical analyses

Group comparisons used parametric tests. When significant interactions were found the simple effects for each group were analysed as recommended by Winer [27] using the pooled error term. For all analyses, the alpha level of ≤0.05 was treated as significant. For the immediate-early gene analyses, two repeated-measures ANOVAs were carried out: one for the hippocampal subregions and a second for the retrosplenial cortex subregions.

## 3 Results

### 3.1 Lateral mammillary nucleus lesions

As the medial mammillary nucleus is known to be important for spatial memory [see 28] it was important that the lateral mammillary lesions did not encroach into the medial mammillary nucleus. The lesions were, therefore, deliberately discrete and none of lesions extended into the medial mammillary nucleus (Fig. 2). However, it was also important that there was considerable cell loss in the lateral mammillary nucleus in order to meaningfully interpret any null effects and it was also necessary for this cell loss to be apparent in both hemispheres [2]. Therefore, five cases that had appreciable unilateral or bilateral LMN sparing were removed from further analyses. The final numbers for the behavioural task were: LMN lesion rats (LMNx), n = 7 and surgical controls (Sham), n = 7. Due to complications unrelated to the experiment, two surgical shams had to be put down prior to the immediate-early gene study, leaving the final numbers as surgical controls (Sham), n = 5, for this part of the experiment.

### 3.2 Radial-arm maze task

**Fig. 3:**
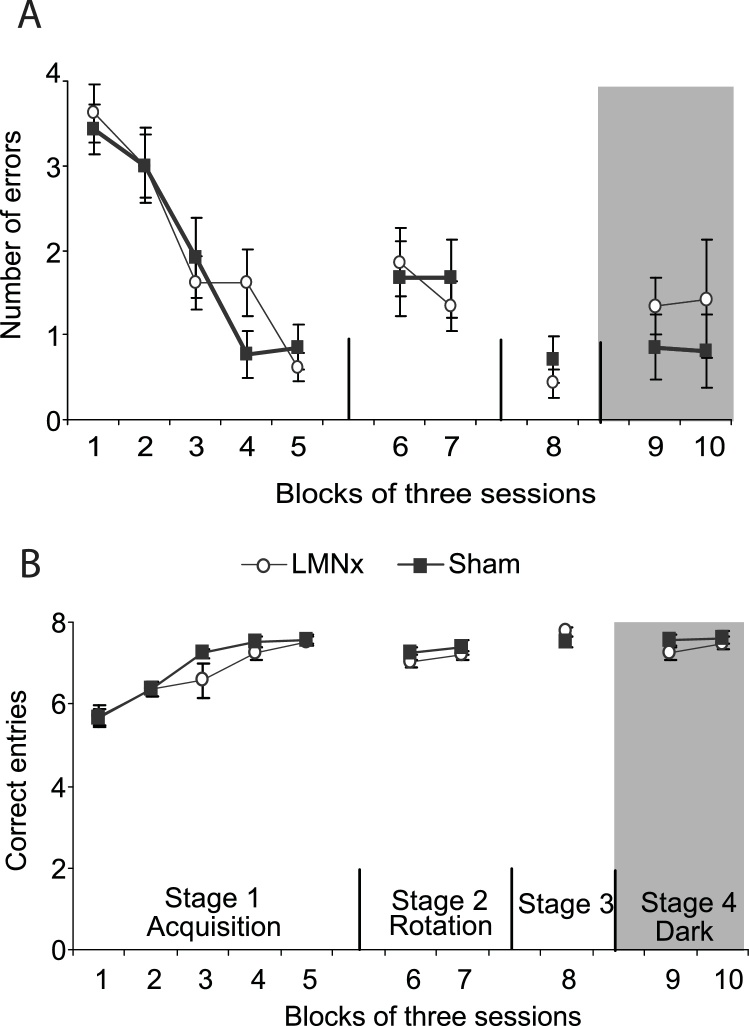
Radial-arm maze task. A. The number of correct entries out of first eight choices for Stage 1 (acquisition), Stage 2 (rotation after first four arm choices), Stage 3 (re-acquisition), Stage 4 (dark) B. Mean number of errors for Stages 1–4. Error bars represent standard error of mean.

Animals were tested on the working memory version of the task where for each session they were required to visit all eight arms of the radial-maze; re-entering a previously visited arm was counted as an error. In addition to the number of errors made, the number of correct arm visits in the first eight choices was also recorded. There was no difference between the lesion and sham groups in the number of errors made during acquisition of the radial-arm maze task or in the number of correct entries in first eight choices (both *F <* 1; Fig. 3). Both groups improved equivalently with training as shown by a significant effect of session (errors, *F*_(14,168)_ = 8.50, *p <* 0.0001; entries, *F*_(14,168)_ = 9.18, *p <* 0.0001) and no group x session interaction (entries, *F*_(14,168)_ = 1.30, *p* = 0.21; errors, *F <* 1). Following acquisition of the task, rats were tested with maze rotation to control for use of intra-maze cues, followed by re-testing on the standard task, then the final stage was testing in the dark (without maze rotation). The same pattern of results was present for all stages as there was no group difference using either error or entry measurement, no effect of session and no group x session interaction (greatest *F* = 1.92; Fig. 3).

### 3.3 Immediate-early gene study

An analysis of the raw cell counts for the septal hippocampus (dentate gyrus, CA3 and CA1; Fig. 1B) revealed no group difference for any of the subfields (all *F <* 1). There was an overall lesion effect for retrosplenial cortex (*F*_(1.10)_ = 11.8, *p* = 0.006; Fig. 1C) as well as a significant group x region interaction (*F*_(2.20)_ = 3.80, *p* = 0.04; Fig. 1C). Analysis of simple effects found this interaction to be driven by a significant lesion-induced reduction of Fos-positive cells in Rgb (*F*_(1.10)_ = 11.5, *p* = 0.007), a borderline effect in Rdg (*F*_(1.10)_ = 5.0, *p* = 0.050) but no significant effect in Rga (*F*_(1.10)_ = 3.53, *p* = 0.09).

## 4 Discussion

In the present study rats with lesions of a central structure within the head direction system, the lateral mammillary nuclei, were tested on a working memory task in a radial-arm maze. This spatial memory task is sensitive to lesions of the hippocampus, anterior thalamic nuclei, mammillary bodies and mammillothalamic tract [8], [25], [29], [30], [8], [25], [29], [30], and the impairments following these lesions could reflect the loss of head direction information. Furthermore, because this task requires animals to distinguish between arms that are radially oriented, it should be particularly sensitive to loss of the head direction system [12]. However, perhaps somewhat surprisingly, the lateral mammillary lesions did not affect performance on the standard working memory task. Rats can adopt non-spatial strategies to solve these types of memory tasks, such as using intramaze cues (e.g. scent trails), which can sometimes mask impairments. This was not the case with the lateral mammillary lesion rats as they were still unimpaired when the maze was rotated mid-way through testing to increase reliance on extramaze cues. Finally, rats were tested in the dark which should encourage rats to use internally generated directional cues to perform the task, however, the lesion rats continued to perform equivalently to their surgical controls when this manipulation was used.

There is some evidence that head direction cell firing corresponds to behaviour in the radial-arm maze. Mizumori and WIlliams trained rats on a radial-arm maze task in the dark and found that with increased training, firing of head direction cells in the lateral dorsal thalamic nucleus showed greater directional specificity and this corresponded with improved performance on the task [32]. While Dudchenko and Taube did not find the same changes in directionality during the acquisition of their radial-arm maze task, they did find correlations between head direction cell firing and performance: a shift in a head direction cell’s preferred direction correlated with a shift in animal’s arm choices [33]. There were, however, task differences between the current study and that reported by Dudchenko and Taube, who used a reference memory task with only one baited arm and a single salient cue was used to direct behaviour. While these electrophysiology studies suggest a correlation between behaviour and head direction cell firing, it would appear from the present study that this may not be critical for performance of a working memory task in the radial-arm maze task.

It is not the case that the lateral mammillary lesions were ineffective: these same rats were impaired on two additional spatial tasks, which have been reported previously [6]: a geometric task in the watermaze and a modified version of the T-maze alternation task. For the T-maze task, there was no impairment on the standard version (see also [5]) but an impairment became apparent when the sample and test phases were carried out in two parallel mazes in order to reduce the use of intramaze cues. This might appear to contradict the present results, as no impairment was found on the radial-arm maze task when the maze was rotated to reduce the use of intramaze cues. A key difference, however, is that in the T-maze task two separate adjacent mazes are used and the sample and test phases are carried out in different mazes. This potentially creates additional confounds, for example, the animals may no longer consider the test trials in the same way given they are now in a new maze. It is also possible that the representation of the mazes in two separate spatial locations may add to the poorer performance. Consistent with this, the lateral mammillary lesion rats were no longer impaired when tested in the two mazes in the dark, which would remove much of this conflicting information. The finding that lateral mammillary lesions animals are unimpaired on performing either the T-maze alternation task in the dark, or the radial-arm maze task in the dark, suggests mammillary nuclei lesions are able to use directional cues to perform spatial memory tasks.

Despite not showing any impairment on the radial-arm maze task, the lateral mammillary lesions in the present study *did* reduce retrosplenial c-Fos levels. The retrosplenial cortex appears to be particularly sensitive to distal damage within the extended Papez circuit, even when the lesioned brain regions do not directly innervate the retrosplenial cortex [24], [25]. Lesions to several different brain regions have been shown to reduce retrosplenial c-Fos activity, these include hippocampus, anterior thalamic nuclei, mammillothalamic tract, and Gudden’s ventral tegmental nucleus [16], [24], [25], [24], [25]. The decrease in c-Fos following lesions to these other regions is typically much greater than that seen following lateral mammillary lesions. In the present study, there is a 25% decrease in overall retrosplenial c-Fos, with the most marked change in granular b, a borderline change in dysgranular and no change in granular a. This can be compared with hippocampal, anterior thalamic and mammillothalamic tract lesions where there is often over 50% reduction in Fos-positive cells and a significant decrease is typically found across all retrosplenial subfields [16], [24], [25], [26], [34]. It is not, however, the case that all lesions, irrespective of location, reduce retrosplenial c-Fos levels. No changes are found following lesions of the entorhinal cortex [26], postrhinal cortex [16] or laterodorsal thalamic nuclei [34] and in contrast amygdala lesions result in an increase in retrosplenial c-Fos levels [24].

The retrosplenial cortex is important for spatial memory [8], [25], [29], [30], [31], [35]. It is, therefore, possible that the reduced c-Fos activity in the retrosplenial cortex contributes to the spatial memory impairments found following lesions to the hippocampal-diencephalic network. However, the present findings would suggest that small decreases in c-Fos activity do not render the retrosplenial cortex completely dysfunctional, given rats with retrosplenial lesions were impaired on all stages of the radial-arm maze task when tested on the same behavioural protocol as used in the present study [36]. Further evidence of a mismatch between retrosplenial c-Fos activity and behavioural performance comes from a study involving anterior thalamic lesions, which markedly reduce levels of retrosplenial c-Fos. Following environmental enrichment, the anterior thalamic lesion rats show an improvement on spatial memory tasks but this is not accompanied by an increase in retrosplenial c-Fos levels, i.e. the levels of c-Fos remain very low [37]. Again, this suggests that retrosplenial c-Fos levels in lesion animals may not closely mirror spatial memory ability.

In contrast to the changes in retrosplenial c-Fos following lateral mammillary lesions, no changes were found in the septal hippocampus. Reduced hippocampal c-Fos has, however, been found following both anterior thalamic and mammillothalamic tract lesions [24], [25]. This dissociation further highlights the differences between the medial and lateral mammillary systems [38]. Although no study has selectively removed just the medial mammillary nucleus, there are reports of clear spatial memory deficits resulting from lesions that principally involved the medial nucleus [39], [40], [41], [42], [43], [44]. Furthermore, cutting the mammillothalamic tract, which can completely disconnect the medial mammillary body projections to the anterior thalamic nuclei while sparing many of the more peripheral lateral mammillary body projections [24], produces very similar behavioural deficits to those seen after complete mammillary body lesions [29], [45], including impairments on the radial arm maze task. As described earlier, mammillothalamic tract lesions produce much greater c-Fos depletions than lateral mammillary lesions [46] and these changes are often similar to those seen after anterior thalamic lesions [17]. Taken together, these findings suggest that the memory systems associated with the medial and lateral mammillary nuclei can be dissociated and that many of the effects of anterior thalamic lesions on distal structures reflect the loss of their medial mammillary body inputs. In contrast, the lateral mammillary nucleus and head direction system, but not the medial mammillary nucleus, appears important for learning about geometric features [6], [25], [47].

It is possible that given the length of time since surgery there could have been some recovery of function, which is why no deficit was observed. Where deficits have been observed following lateral mammillary body lesions, the typical pattern is an initial impairment (i.e. over the first few days of testing) but by later test sessions the lesion animals are performing at equivalent levels to their surgical controls [5], [6]. While recovery of performance within tasks has been found, this does not appear to carry over to subsequent behavioural tests. For example, it is not the case that impairments are only found on the first behavioural task that animals are tested on post-surgery. Furthermore, lesions to related regions, such as the mammillothalamic tract, still impair performance on the radial-arm maze task after similar testing protocols, again highlighting the difference between medial and lateral mammillary projections [25]. Irrespective of the post-surgery interval and testing protocol, an important finding is that the lack of behavioural impairment was still accompanied by a reduction in retrosplenial c-Fos.

Together, these results show that the head direction system does not appear to be necessary for the acquisition of a task that one might presume to be heavily reliant on the head direction system. There are still remaining questions regarding the exact role of the head direction system for spatial memory and navigation. It clearly does contribute to spatial memory, for example in geometric learning [6] and rapid learning of new locations [5], [7] but it may be that it is less important when tasks are learnt incrementally. The reductions in c-Fos highlight the sensitivity of the retrosplenial cortex to distal damage within the hippocampal-diencephalic network. Nonetheless, given the lesion animals were unimpaired on the radial-arm maze task, this would suggest that a small decrease in retrosplenial c-Fos is not sufficient to result in behavioural impairments and does raise questions about how closely c-Fos levels predict functional outcomes.

## Funding

This work was supported by a Wellcome Trust Senior Research Fellowship (grant number WT090954AIA)
